# Non neovascular Age – related macular degeneration – Review on clinical and imaging advances

**DOI:** 10.1007/s00417-026-07216-1

**Published:** 2026-03-30

**Authors:** Maria Cristina Savastano, Claudia Fossataro, Lorenzo Hu, Francesco Mottola, Nicola Claudio D’Onofrio, Valentina Cestrone, Federico Giannuzzi, Stanislao Rizzo

**Affiliations:** 1 Department of Ophthalmology, Catholic University of the Sacred Heart, Rome, Italy; 2 Department of Ophthalmology, Fondazione Policlinico Universitario Agostino Gemelli, IRCCS, Rome, Italy; 3 Istituto di Neuroscienze, Consiglio Nazionale delle Ricerche (CNR), Pisa, Italy

**Keywords:** Acquired vitelliform lesion, Drusen, Dry age – related macular degeneration, Hyper-reflective foci, Pigment epithelium detachment, RORA

## Abstract

The aim of this review was to provide an overview of the principal features of non-neovascular age – related macular degeneration (NNAMD) and how they appear by multimodal imaging.

None are specific of the disease, but their simultaneous detection, their distribution and the development in a particular age range allow to make the correct diagnosis. The current availability of different imaging modalities offers the chance to better characterize the disease and to detect the earliest changes, monitoring its progression to the late stage, known as geographic atrophy (GA). A recent consensus has provided a novel definition of GA, based on optical coherence tomography (OCT), introducing two novel concepts: incomplete and complete retinal pigment epithelium (RPE) and outer retinal atrophy (iRORA and cRORA, respectively). The main recognized risk factors for progression are soft drusen, drusenoid pigment epithelium detachment (PED), subretinal drusenoid deposits (SDD), intraretinal hyperreflective foci (iHRF) and acquired vitelliform lesion.

Soft drusen represent one of the earliest signs of NNAMD and their confluence can lead to the development of drusenoid PED, whose height mainly correlates with progression. SDD have been better characterized after the advent of the OCT and differently from the drusen, the material accumulates above the RPE.

iHRF could be observed in different retinal layers, but the involvement of the outer nuclear layer has been recognized to correlate more with GA development.

The identification of valid biomarkers is crucial to monitor and predict the disease progression and to evaluate the efficacy of novel proposed therapies in clinical trials.

## Key messages

***What is known:***

- Dry age-related macular degeneration (AMD) is the major cause of irreversible central vision loss in the elderly and its diagnosis is typically made by fundus examination, fundus autofluorescence (FAF) and optical coherence tomography (OCT) scans.
  In everyday clinical practice, multimodal imaging allows for the identification of several biomarkers extremely useful not only for the diagnosis, but also to monitor the disease progression and to predict its evolution.

***What is new:***

- This review aims to focus on the main findings of dry AMD referring to the most important and used imaging modalities.
- Particular interest has been given to the role of OCT to highlight the most used biomarkers, recognized also by the CAM group.

## Introduction

Age – related macular degeneration (AMD) is the major cause of central vision loss in the elderly in industrialized countries [1, 2]. It has been estimated that about 200 million people around the world suffer from this condition and, in light of population aging, a markedly increase, reaching 288 million of patients in 2040, is expected. European countries currently show the highest percentage of cases, although in the near future, Asia, accounting about half of worldwide population, could most likely take on the first place [3]. 

The disease is typically distinguished in two main categories: the most common, non neovascular AMD (NNAMD), representing around 80% of cases, and neovascular AMD (NVAMD), which threatens more severely and rapidly the central vision.

AMD is a multifactorial disease, in which genetic predisposition combined to several and various environmental factors, mainly ageing and cigarette smoking, plays a crucial role in its onset and progression. It seems that other systemic co-factors such as arterial hypertension, high-density lipoproteins (HDL) and cholesterol, body mass index (BMI) could increase therisk of developing the disease [4–7]. 

At the beginning, patients do not complain about any symptoms and only few retinal changes are detectable at fundus examination, mainly involving the retinal pigment epithelium (RPE). These consist in small or large drusen deposits and pigmentary abnormalities, which are represented in higher proportion than expected because of age. As the disease progresses, detachment of the RPE and thinning of the outer retinal layers can also be observed. The natural history of AMD could be then complicated by the development of macular neovascularization (MNV), pathognomonic of NVAMD, or could further progress and evolve to geographic atrophy (GA). In a small proportion of cases, the two subtypes could also co-exist. When the disease reaches the most advanced stages, patients’ quality of life is compromised due to invalidating symptoms such as central vision loss, central scotoma, metamorphopsia, binocular vision impairment, which significantly impair the everyday-life’s activities [8]. 

The great innovations in the multimodal imaging field have allowed a more accurate knowledge of AMD, identifying novel biomarkers and providing significant insights into its pathogenesis. The above-described schematic staging method has been replaced by new classifications that take into account the role of different findings in the prediction of disease progression. One example is the updated Age-Related Eye Disease Study (AREDS) simplified severity scale, which focused on the role of reticular pseudodrusen (RPD) and proposed an updated definition of “late AMD” [9]. 

RPD, also known as subretinal drusenoid deposits (SDD), have been recognized as a negative biomarker related to late AMD, independently from soft drusen and pigmentary abnormalities, doubling the risk of progression.

Furthermore, the updated scale redefined the outcome of late AMD, including non-central GA besides neovascular AMD and central GA. This remarkable change has reflected the modern clinical practice, where noncentral GA is considered a significant endpoint due to its inevitable involvement of the macula [9]. 

The great steps forward in the comprehension of the NNAMD have paved the way for new research in the therapeutical field. On February 2023, the Food and Drug Administration (FDA) approved Pegcetacoplan and Avacincaptad Pegol which, targeting the C3-C3b factors and the C5 factor, respectively, regulate the complement system, with the aim of slowing down the progression of atrophy and promoting the photoreceptors survival [10, 11]. 

However, Pegcetacoplan has not been approved by European Medicines Agency (EMA) in September 2024, because considered scarcely beneficial and risky for patients in view of the possible inflammatory and neovascular adverse events [12, 13]. 

A novel therapeutical approach has been recently proposed for intermediate AMD patients, consisting in lutein administration by scleral iontophoresis, aiming to prevent the progression to GA [14]. Rinaldi et al. showed an increase in macular retinal pigment and an improvement in retinal sensitivity in treated eyes [15]. 

Photobiomodulation (PBM), based on the use of light (wavelength 500–1000 nm), seems to stimulate the mitochondrial respiratory chain, promoting the ATP production. Its application has been evaluated in early-intermediate AMD eyes, demonstrating a significant gain in visual acuity, no increase in drusen volume and less onset of GA lesions in treated eyes compared to sham [16, 17]. 

Recently, our group has proposed the use of subretinal cord-blood platelets rich plasma (CB-PRP) injection in GA patients, in order to decrease its progression rate, providing trophic and regenerative factors [18]. Moving forward, we evaluated the safety and efficacy of repeated intravitreal injections of CB-PRP, assessing a significant slowing down in GA extension [19]. 

The aim of this review was to provide a comprehensive perspective on the main biomarkers of NNAMD, emphasizing their imaging characteristics across different diagnostic imaging modalities and to provide the essential tools for diagnostic evaluation and longitudinal monitoring of the disease.

## Drusen

Focal-yellow, white deposits of extracellular debris, lying between the basement membrane of the RPE and the inner collagenous zone of Bruch’s Membrane (BM), known as drusen, are considered the clinical hallmarks of AMD [20]. Of note, drusen are typical of AMD, but they could be observed in otherwise healthy elderly people and in different pathological conditions like chronic retinal detachment, membranoproliferative glomerulonephritis II, choroidal pigmental lesions and other retinal dystrophies.

Drusen were first described as “Colloidkulgen” in 1855 by Donders referring to their sphere reflective appearance surrounded by rings of hyperpigmentation [21]. Anatomische further defined them as *“geode”* based on their yellow and shiny feature at fundus ophthalmoscopy [22]. 

Drusen can develop with ageing and due to oxidative stress combined with inflammatory stimuli and abnormal lipid metabolism, leading to BM thickening, choroidal capillary atherosclerosis and RPE dysfunction [23]. Several genes mutation and dysregulation such as CFH, complement CR, APOE, toll-like receptor 4 (TLR4), ARMS2, HTRA1 are responsible at the biomolecular level of drusen formation [20]. Histochemical and immunohistochemical studies have shown that drusen include a variety of polysaccharides, glycosaminoglycans and glicoproteins such as tissue inhibitor of metalloproteinase 3, ubiquitin, vitronectin, zinc, ApoE and activation fragments of complement C [24]. 

As a consequence of drusen accumulation, the altered RPE-BM-choroidal complex become not able to fulfill its role of photoreceptor outer segments phagocytizing, with further accumulation of extracellular deposits [25]. Furthermore, drusen, separating the RPE from the BM-choroidal complex, significantly impair the supply of oxygen and choroidal flow, leading to migration of RPE cells, as the last attempt of survival, or to RPE cells death [25]. 

**Fig. 1:**
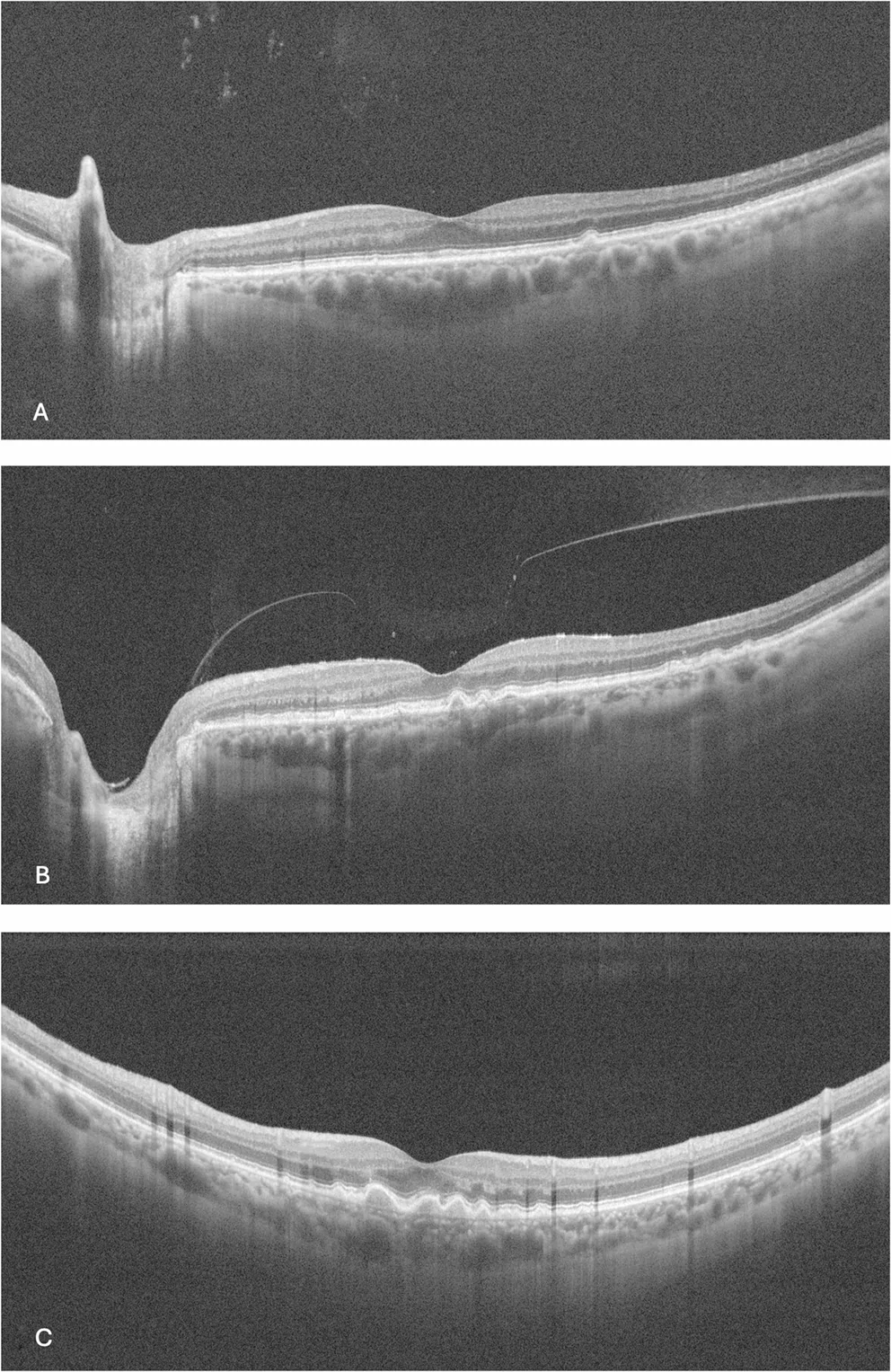
Optical coherence tomography (OCT) and drusen: Optical coherence tomography (OCT) B – scans showing hard drusen (1 **A**), intermediate (1**B**) and soft drusen (1 **C**)

The Wisconsin Age-Related Maculopathy Grading System, the International Classification system and the AREDS classification system refer to *“hard drusen”*, if the diameter is lower than 63 micron (this diameter corresponds to the half of the width of a large vein at the optic disc margin), *“intermediate drusen”*, if the diameter is between 63 and 125 micron, and *“soft drusen”*, if larger than 125 micron (Fig. 1) [26]. 

However, beyond this schematic classification, optical coherence tomography (OCT) studies and histological analysis have given the chance to distinguish other subtypes: cuticular drusen, calcified drusen and pseudodrusen [27]. 

The characterization of size, number and appearance of drusen, in association with pigmentary changes defines the stage of AMD and enables to predict the future evolution to MNV or GA. Color fundus photography (CFP) plays a significant role in the diagnosis and follow up of drusen, allowing to detect the increase in number and dimension as well as their widespread distribution over time.

By fundus autofluorescence (FAF), drusen may result hypo-, hyper or even isoautofluorescent compared to the normal background, although larger deposits are usually associated with signal changes. RPD show a pathognomonic FAF pattern, characterized by roundish spots with decrease intensity, surrounded by normal FAF signal. On OCT – angiography (OCTA), focal thinning of choriocapillaris could be appreciated underneath the drusen and the flow deficit tends to progress with consequent loss of the vascular structure when atrophy develops [28]. 

### Hard drusen

Hard drusen represent the drusenoid deposits and are considered a normal consequence of ageing, even though they could even be found in younger people. However, the presence of drusen, especially in a considerable quantity, should be regarded as the onset of AMD disease. Although the presence of several hard drusen could be a heritable condition associated with higher risk of developing AMD, this misses a strong correlation with proven AMD genotype [29]. In fact, CFHY402H polymorphism is exclusively associated with drusen larger than 63 micron. Moreover, CFH mutation and APOE E2 genotype, involved in the AMD pathogenesis, are not directly related to hard drusen development [30]. Hard drusen appear as small (diameter less than 63 micron), yellow-white, round deposits with distinct edges [31]. Formation of hard drusen is usually preceded by focal densifications of BM, known as microdrusen, made of dense amorphous material. Indeed, hard drusen tend to cluster and fuse over a row of microdrusen.

As long as the rim is intact, clustered hard drusen are related with good visual prognosis, although their accumulation in the fovea may be associated with slight symptoms. Usually, confluent drusen degenerate over time, leaving foci of thinned and atrophic RPE and sub-RPE globular deposits.

Histologically, drusen appear eosinophilic with a compact structure in comparison with soft drusen and a uniform color density from their center to the edge. Despite drusen can be found either in macula or peripheral retina, they show few differences. Macular drusen are usually larger and have a more heterogenous structure, with higher probability to calcify, than peripheral ones [32]. 

In spite of their high phospholipid content proportions, hard drusen show mild hyperfluorescence both on fluorescein angiography (FA) and indocyanine green angiography (ICGA). On OCT B scan, small dome shape irregularities of the RPE could be evident, without interruption of the above ellipsoid zone (EZ) and external limiting membrane (ELM).

### Soft drusen

Soft drusen or basal linear deposit (BLinD) are extracellular lipid rich deposits which accumulates on the BM, considered pathognomonic of AMD [33]. The major component is esterified cholesterol and lipoprotein, mostly apoB and apoE secreted by RPE in its functional cycle.

In comparison to hard drusen, soft ones usually appear larger (> 125 micron) in diameter and with less demarcated borders. Their pale-yellow color is characteristic, and it can be an important hint to differentiate them at the fundus exam from hard drusen. Moreover, they can be divided in “soft distinct”, if they have a uniform density, nodular surface and sharp margin or “soft indistinct”, if they have a decreasing density from center to periphery with fuzzy edges [34]. 

Soft drusen are usually found at the posterior pole (more frequently in central macula, temporal and superior quadrants), and with SDD, represent the two major factors related with increased risk of progression to advanced AMD [35]. 

By OCT, soft drusen appear as dome-shaped RPE hyperreflective elevations with sloping sides that can range from 30 to 1000 micron [25]. 

The drusen volume and size are predictors for regional future atrophy: major volume and bigger drusen size increase RPE distance from the choriocapillaris complex and seems to exacerbate the already complex lipotoxic, hypoxic and micronutrient deficient retinal environment [36]. 

**Fig. 2:**
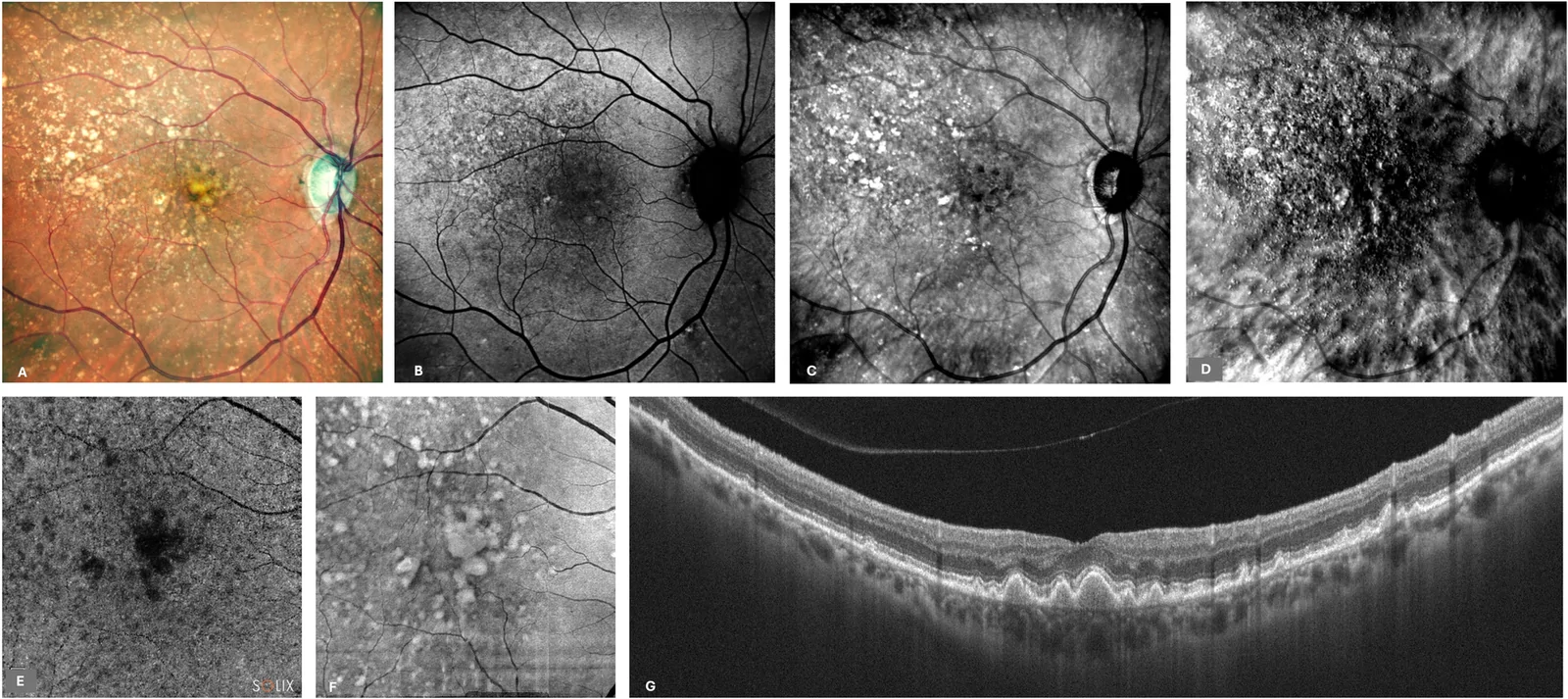
Multimodal imaging of non neovascular – age related macular degeneration (NNAMD) patient with soft drusen. Color fundus photography (CFP) show at the posterior pole several yellowish lesions, corresponding to soft drusen with foveal involvement (2 **A**). These lesions appear hypo/hyperautofluorescent on fundus autofluorescence (FAF) and hypo/hyperreflective on Infrared (IR) (2**B** – **C**). At retromode (RM) imaging, the drusen appear as elevated lesions, rendering a rough aspect to the retina (2**D**). Soft drusen are responsible of choriocapillaris flow voids as appreciable from optical coherence tomography (OCT) – angiography (OCTA) (2**E**). The enface shows the hyperreflective lesions in the macular region which correspond to multiple drusenoid retinal pigment epithelium (RPE) humps on OCT B scan, associated with few cuticular drusen (2 **F** – **G**)

Figure 2 illustrates multimodal imaging of soft drusen.

### Cuticular drusen

**Fig. 3:**
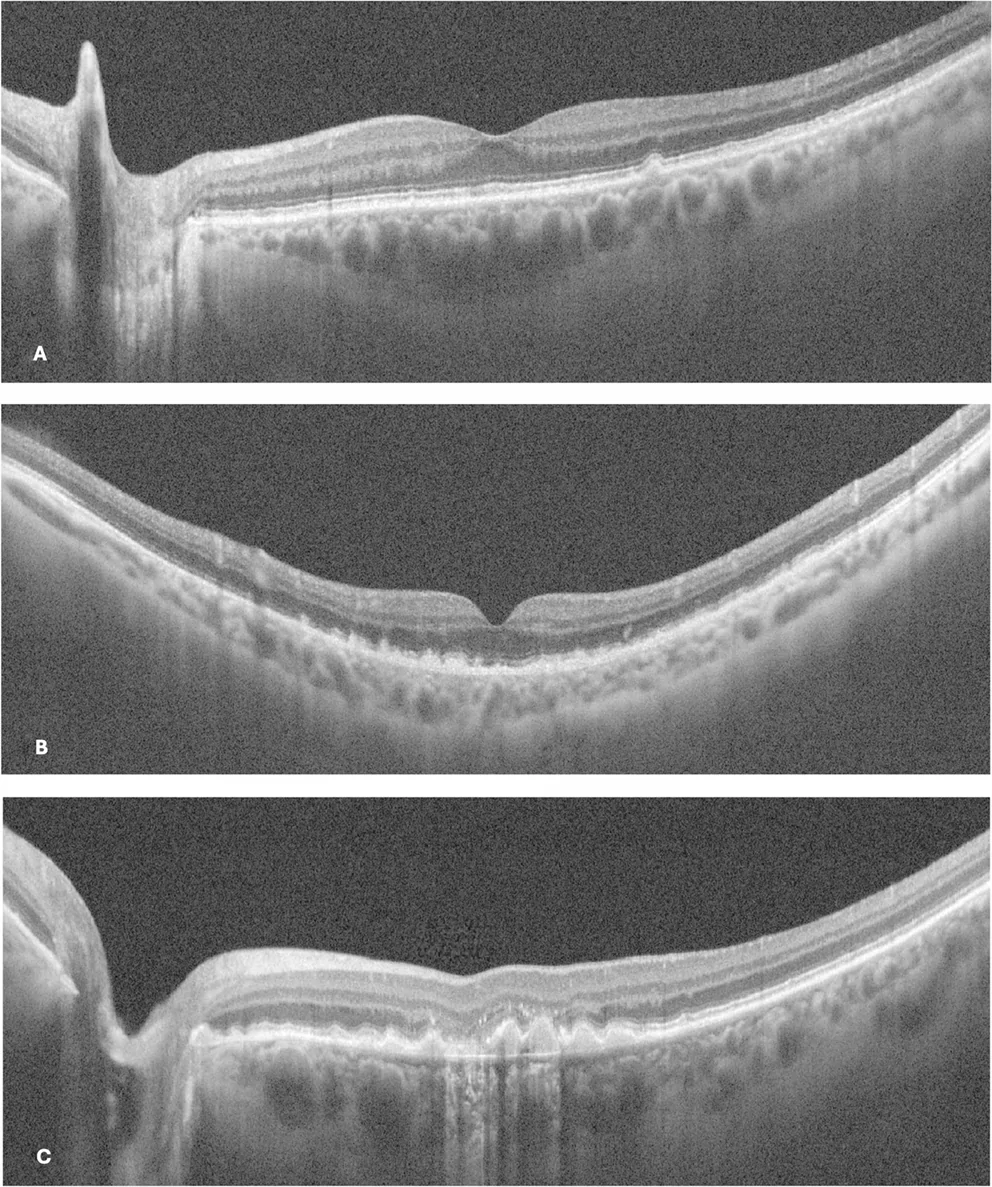
Optical coherence tomography (OCT) and cuticular drusen: OCT B scans illustrate different phenotypes of cuticular drusen. 3 **A**) The OCT scan shows only one small soft drusen associated with cuticular drusen. 3**B**) The image shows the prevalence of cuticular drusen with only retinal pigment epithelium (RPE) remottling. 3 **C**) Cuticular drusen are associated with soft drusen (> 200 micron) and a focal area of outer retina and RPE atrophy highlighted by the signal hypertrasmission

Cuticular drusen were first described by Gass in 1977 as small (25–75 micron) dot-like yellow, round subretinal deposits clustering in macula or peripheral retina. Based on their localization, cuticular drusen have been distinguished in three phenotypes (Fig. 3): [37]

Phenotype 1: cluster of small lesions concentrated in the macular region and rarely extending beyond vascular arcades;

Phenotype 2: scattered lesions extending into the peripheral retina;

Phenotype 3: cuticular drusen associated with large drusen (> 200 micron);

Nowadays, cuticular drusen, especially phenotypes 2 and 3, are considered significant risk factors for progressing to advanced AMD, more likely to GA rather than MNV.

In the past, these lesions were thought to be the consequence of nodular thickening of the basement membrane of the RPE and thus named “basal linear deposits”. Histological analysis demonstrated that these lesions share with conventional drusen the localization between RPE-basal lamina (BL) and BM [38]. Cuticular phenotype has a female preponderance, a strong association with single nucleotide polymorphism on the CFH gene and a possible association with SDD [39]. Beyond the field of AMD, cuticular drusen could also be associated with membranoproliferative glomerulonephritis (MPGN) type II and acquired vitelliform lesion (AVL).

Cuticular drusen have been also described as *“stars in the sky”* for their appearance during the arteriovenous phase in FA, for their typical central hypoautofluorescent core and hyperautofluorescent rim in FAF due to RPE erosion [40]. 

Although sharing the same content of conventional drusen, cuticular ones can be distinguished by hard drusen because of their higher number that facilitates their identification in CFP. However, over time as drusen accumulate and coalesce, cuticular subtype can be mistaken for soft drusen. Enface OCT could provide a significant support in their differentiation. Soft, cuticular and calcified drusen at the RPE slab (30–70 micron above the BM) in enface OCT shows a hyporeflective core with hyperreflective margin, defined as “donut sign”. In order to distinguish the three subtypes, it is necessary to scroll the analyzed slab to the choroidal level (0-400 micron below the BM), where soft drusen appear isoreflective or slightly hyporeflective, cuticular drusen becomes reverse donut with hyperreflective core and hyporeflective borders, calcified drusen appear as dark lesion [41]. 

Similar to soft drusen, cuticular drusen’s lifecycle is characterized by growth, coalescence and resorption, leading to RPE abnormalities and subsequent macular complication.

### Calcified drusen

Calcified drusen were reported to contain calcific nodules on histopathological examination. However, finding of hydroxyapatite in all subretinal deposits supports the theory by which all drusen are calcified just in different degree. Indeed, as drusen regress with chronicity, their calcium content may increase [42]. 

By this, calcified drusen are not considered to be a specific drusen subtype, but just a measure of their age.

**Fig. 4:**
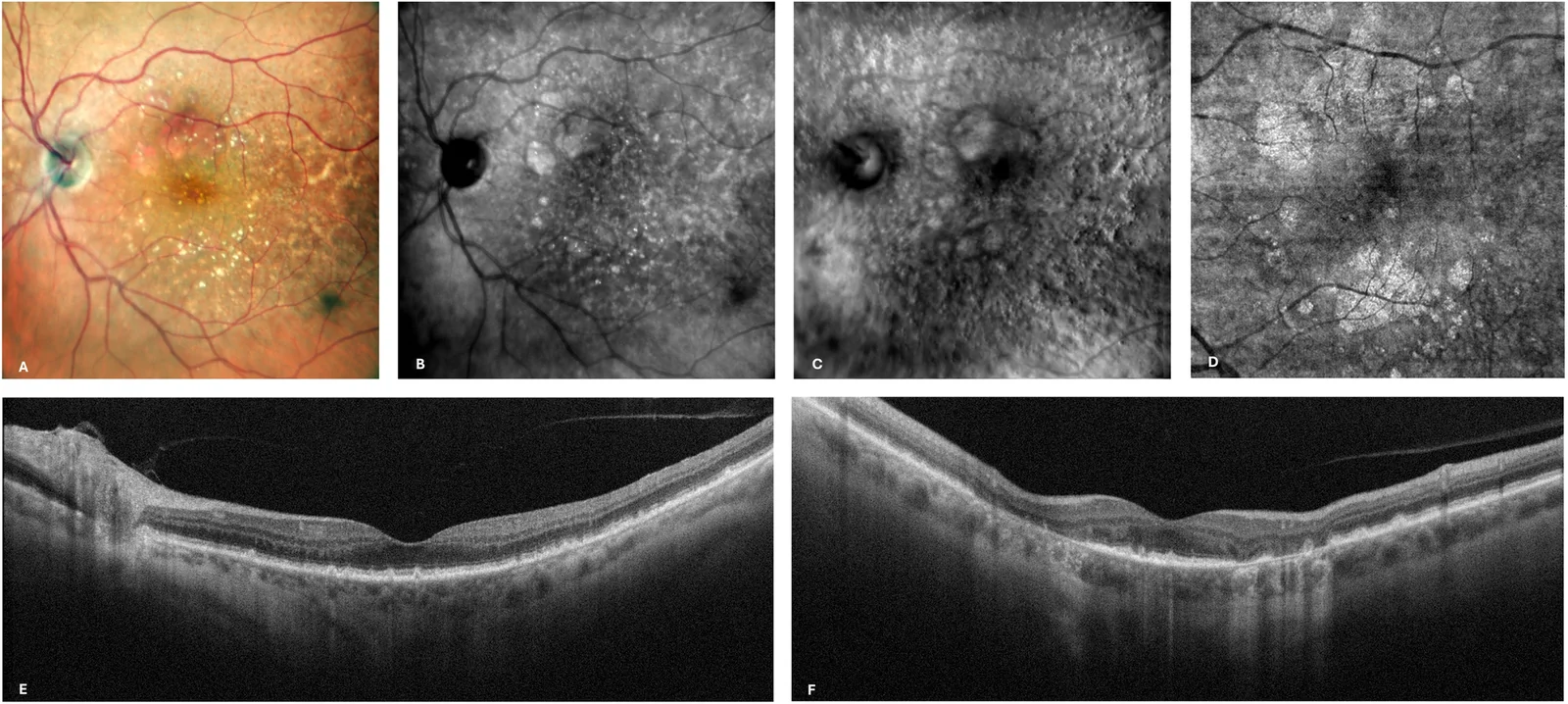
Multimodal imaging of a non neovascular age – related macular degeneration (NNAMD) patient with calcified drusen. Color fundus photograph (CFP) shows several drusen at the posterior pole, associated with calcified drusen, which appear as whitish lesions surrounding the fovea (4 **A**). Focal areas of atrophy are evident superiorly to fixation, which appear well-demarcated in Infrared and retromode (RM) imaging (4 **A** – **C**). Enface Optical coherence tomography (OCT) reveals an additional area of atrophy, inferior to the fovea (4**D**). Horizontal and vertical OCT scans passing though the fovea show the pointed calcified drusen and the signal hypertrasmission around the fovea associated with outer retinal tubulation and subsidence of inner nuclear layer (INL) and outer plexiform layer (OPL) nearby the residual retinal pigment epithelium (RPE) and ellipsoid zone (EZ) layers (4**E** –** F**)

Calcified drusen resulted hypoautofluorescent, but to which extent, depends in their topographical location: central calcified drusen lead to a larger hypoautofluorescent shadow than the peripherally placed drusen (Fig. 4). By OCT, they appear with heterogeneous inner reflectivity or with hyporeflective core and hyperreflective border.

### Pseudodrusen

In 1990, Mimoun et al. used the expression “*pseudodrusen visible en Lumière blue*”, referring to their strong visualization in blue light, to describe retinal lesion of 100 micron size that appeared yellowish in CFP but not hyperfluorescent in FA [43]. 

Furthermore, Smith et al. coined the term “reticular macular disease” and Zweifel et al. favored the term SDD. Nowadays, it’s preferable to use the term “RPD” when referring to retinal lesions in the context of AMD, whereas “SDD” when describing their topography. SDD may be observed even in association to other retinal disease such as pseudoxanthoma elasticum, Sorbsy’s dystrophy, IgA neuropathy, Vitamin A deficiency, fundus albipunctatus, and adult onset foveomacular dystrophy [44–49]. 

RPD have also been associated with a recent described entity, known as extensive macular atrophy with pseudodrusen-like appearance (EMAP) [50]. Differently from GA – AMD related, the macular atrophy in EMAP patients extends typically along the vertical axis and shows multilobular borders. The RPD are visible at the posterior pole and in the mid-periphery before the onset of macular atrophy and pavingstone-like lesions [51]. In the latest stage, the mid-periphery atrophic areas could coalesce with the macular atrophy, usually sparing a temporal island of healthy retina.

SSD size ranges between 125 and 250 micron, they appear whiter than soft drusen and contrary to them, they are rich in unesterified cholesterol and lack of apoB, apo1 and esterified cholesterol.

SSD are predominant in increased age, in female sex, likely due to the higher prevalence of systemic autoimmune inflammatory disease, and they are more often found bilaterally than unilaterally [52]. 

According to their predilection toward rods-photoreceptor, usually, RPD are found in the superior perifoveal region, with the highest density, followed by temporal and inferior outer macula area [53]. Due to this characteristic distribution, scotopic defects and impaired dark-adaptation represent the main complained symptoms. Furthermore, Voichanski et al. demonstrated a trizonal distribution pattern of drusen and SDD using enface OCT scans by overlapping the ETDRS grid: macular drusen resulted to be focused in the central circle, while dot SDD are mostly found in the inner ring and in the inner part of the outer ring, and finally ribbon SDD localized typically in the outer ring of the grid or outside it, with potential involvement of the retinal periphery [54]. Surprisingly, this particular distribution matched with the rods and cones density [55]. 

Zweifel et al. produced a tree-stage classification of RPD appearance in OCT. In stage 1, diffuse hyperreflective material accumulates between the RPE and the EZ. In stage 2, the accumulation goes on enough to deform the contour of the EZ. In stage 3, deposits assume a conical shape that breaks through the EZ [56]. Moreover, Querques et al. added a fourth stage to this classification, characterized by lesions regression and a migration through the inner retinal layers [57]. 

**Fig. 5:**
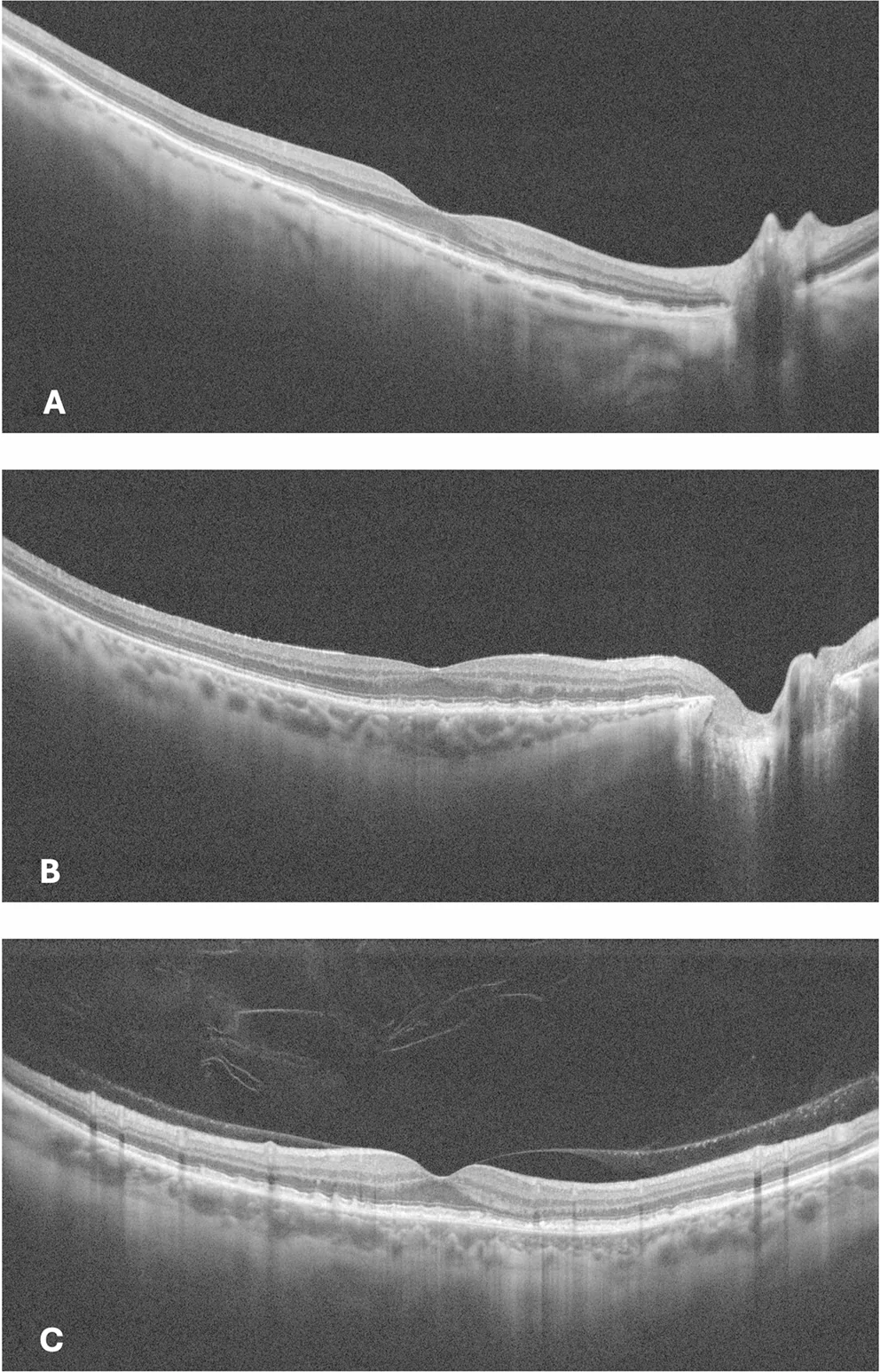
Optical coherence tomography (OCT) and pseudodrusen: OCT B – scans showing reticular pseudodrusen at early stage (5 **A**), intermediate (5**B**) and advanced (5 **C**)

Figure 5 illustrates different stages of pseudodrusen on OCT.

Overall, RPD can be considered a third macular risk factor, other than soft drusen and pigment abnormalities, related with increased risk of progression to late AMD, both MNV and GA. In NVAMD, type 3 MNV have shown to have a particular association with SDD than other types of neovascularization. Furthermore, NVAMD eyes with SDD have a worse prognosis, due to poor response to treatments, greater visual decline and faster GA development [58]. 

## Pigment epithelium detachment

Retinal pigment epithelial detachments (PED), characterized by RPE separation from the underlying BM layer, are common findings of different chorioretinal diseases, but in 76% of cases, they are related to AMD [59]. 

Although PEDs commonly result asymptomatic, few patients may report painless blurred vision, metamorphopsia or induced hyperopia.

Their inner structure is responsible of the OCT aspect, distinguishing three main PEDs types: drusenoid, serous and vascular. Serous PEDs appear hyporeflective in OCT with empty lumen, but they are not characteristic of AMD. Vascular PEDs take this name because of the development of a neovascular network below the RPE, that could complicate the AMD course, converting to NVAMD.

In view of the topic of this review, we focused our attention on drusenoid PEDs.

### Drusenoid PED

Drusenoid PEDs are well-defined, pale yellow or white, elevated lesions of at least 350 μm in the narrowest diameter, deriving from the confluence of many large soft drusen [60]. 

At fundus examination, they are usually confined at the posterior pole with either smooth or wavy borders. Associated pigmentary changes are common, with hyperpigmentation being significantly more frequent than hypopigmentation. A characteristic feature is the appearance of a speckled or stellate pattern of brown or gray pigmentation on their surface. Large soft drusen frequently encircle these pigmentations and may resemble solitary sizable deposit, especially in eyes with abundant and confluent drusen.

In OCT, drusenoid PEDs typically exhibit a well-defined contour corresponding to the elevated hyperreflective RPE, often displaying a smooth appearance. Below the detached RPE, a compact and uniform material with moderate-high reflectivity is typically collected.

Drusenoid PEDs are commonly not linked to the presence of subretinal or intraretinal fluid [61]. 

The PED extension could be appreciated by enface OCT, which clearly highlights the limits between the normal retina and the PED. OCTA is usually sufficient to exclude the development of a neovascular network and only in more challenging cases, such as in case of very high PED, FA could provide a significant support.

On FA, drusenoid PEDs exhibit a subtle hyperfluorescence in the early phases, followed by a gradual increase and stabilization in the late phases without any signs of leakage. A jagged pattern frequently characterizes the borders of the PED. Focal areas of hypofluorescence can be observed, corresponding to the masking effect caused by pigment deposits.

Since they hinder the visualization of choroidal vessels, PEDs appear hypofluorescent in the late phases of ICGA.

Drusenoid PED appearance on FAF is not univocal, since both an increase and a decrease in FAF signal, due to pigment accumulation, could be detected. Additionally at PED margins, a halo of decreased autofluorescence could be visible, finding in common with serous and mixed PEDs, but which is absent in vascular PEDs. Of note, the presence of this halo appears to be independent from subretinal fluid (SRF).

Regarding this feature, Bindewald-Wittich et al. suggested a potential connection with the steepness of the RPE elevation, which is more abrupt in eyes with serous or drusenoid PED compared to vascular ones. They hypothesized that the above retinal layers compensate for the rapid ascent of the RPE, resulting in a smoothly elevated surface with increased retinal thickness, leading to greater absorption of the excitation light and increase in FAF signal within the transition zone. However, it is important to acknowledge that any additional accumulation of SRF overlying the PED can partially or completely disrupt FAF changes attributed to the PED [62]. 

The natural progression of drusenoid PED includes three possible scenario: collapse with consequent progression towards GA, vascularization followed by exudation, or stabilization [63]. 

Eyes with drusenoid PEDs are at greater risk of advancing to GA within 5 years. According to AREDS Report Number 28, at 3 years of follow-up, 17% of eyes progressed to NVAMD, reaching 23% by the 5-year mark [60]. Although the cumulative rate of progression to GA was initially lower during the early phase of follow-up, it was anticipated to rise over time and surpass the cumulative rate of progression to NVAMD by 7 years. Overall, approximately 42% of eyes with drusenoid PEDs advanced to either form of advanced AMD within 5 years—a progression rate significantly higher than that observed in a control group with large drusen and hyperpigmentation, but lacking drusenoid PEDs.

Two aspects of drusenoid PEDs have been related to late stages of AMD, the width and the height of the PED. The former is typically greater in patients who develop NVAMD, while the latter shows higher values in those who progress to GA [64]. 

Moreover, the size of drusenoid PED and onset of related symptoms seem to predict the progression to advanced AMD. Roquet et al. observed that PEDs greater than two-disc diameters (DD) or accompanied by metamorphopsia as initial symptom have a higher probability of progressing to GA or NVAMD after 2 years [65]. 

Drusenoid PEDs are associated with an increased risk of ≥ 3 lines loss and with significant loss in vision (> 15 letters) [64, 66]. It has also been reported a correlation between the increased height of the PED and the increased odds of progression. Additionally, the volume, the maximum diameter, the maximum height and the topographic location of the PEDs were related to lower visual acuity [67]. These findings could be explained considering that higher PEDs experience lower nourishment of the RPE by the underlying choriocapillaris due to the increased distance between the two layers, potentially resulting in disruption of the RPE and consequent collapse of the drusenoid PEDs.

In view of their typical distribution, mainly in the middle and inner rings of the ETDRS-grid, their location has been related to greater visual impairment, but it does not seem to influence the disease progression [67]. 

## Hyper-reflective Foci

Nowadays, intraretinal hyper-reflective foci (iHRF) are considered one of the strongest risk factors of progression from intermediate AMD to the late stage of the disease, unrelated to soft drusen, pigmentary abnormalities and drusenoid PEDs [68]. 

Regarding their pathogenesis, the most validated hypothesis stated that iHRF develop consequently to dissociation of dying or damaged RPE cells towards the deep capillary plexus (DCP) [69]. Verma et al. supposed that choriocapillaris ischemia represents the trigger to RPE cells migration within the DCP.

On the other hand, inflammation or microglia degeneration may be responsible of iHRF in the inner layers [70, 71]. 

On CFP, iHRF are typically responsible of hyperpigmented change.

The analysis of OCT B scan allows to observe their widespread distribution within the retinal layers, although those sited in the outer nuclear layer (ONL) seemed to be prevalently associated with disease progression [72]. It has been demonstrated that iHRF in the ONL increase remarkably over 24 months, while those sited in the inner layer are more stable in number over time.

Mahmoudi et al. observed that EZ disruption, height and width of drusen were independent risk factors for the development of iHRF, as well as the location closer to the fovea [73]. 

## Acquired vitelliform lesion

Vitelliform macular lesion, typically associated with Best’s vitelliform dystrophy, can also manifest in adults as what is termed “acquired vitelliform lesions” (AVL). These AVLs include a wide range of macular pathologies, particularly degenerative conditions such as AMD, dystrophic diseases as adult-onset foveomacular dystrophy (AOFVD), paraneoplastic manifestations, toxic effects (e.g., deferoxamine toxicity), and vitreoretinal interface disorders such as epiretinal membrane and vitreomacular traction [74]. 

In cases where AVL is associated with AMD, patients may exhibit other typical findings of the disease that could support in the diagnosis making process. Histologically, the vitelliform lesion is characterized by the accumulation of various substances such as lipofuscin, melanosomes, melanolipofuscin, and outer segment debris within the subretinal space, above the RPE. A clinicopathological case series conducted on 14 eyes revealed two possible distinct sources for the vitelliform material: an internal source originating from the outer segment discs of the photoreceptors, and an external one originating from the RPE. The propensity of AVLs to occur beneath the fovea suggests a potential association with cones and their supportive cells [75]. 

Recent findings have highlighted a strong association between AVLs and pachychoroid features, including pachyvessels and increased choroidal thickness. A cross-sectional study of 217 eyes found that AVL lesions located over drusen and those with overlying iHRF exhibited greater lesion height and width [76]. This suggests that structural and vascular changes in the choroid may play a role in AVL pathogenesis and progression.

AVLs were initially described by Gass and then by other researchers as solid slightly raised yellow lesions that exhibit hyperautofluorescence on FAF. These lesions typically measure approximately one-third the diameter of the optic disc and are frequently located beneath the fovea, often featuring a pigmented spot [77, 78]. 

**Fig. 6:**
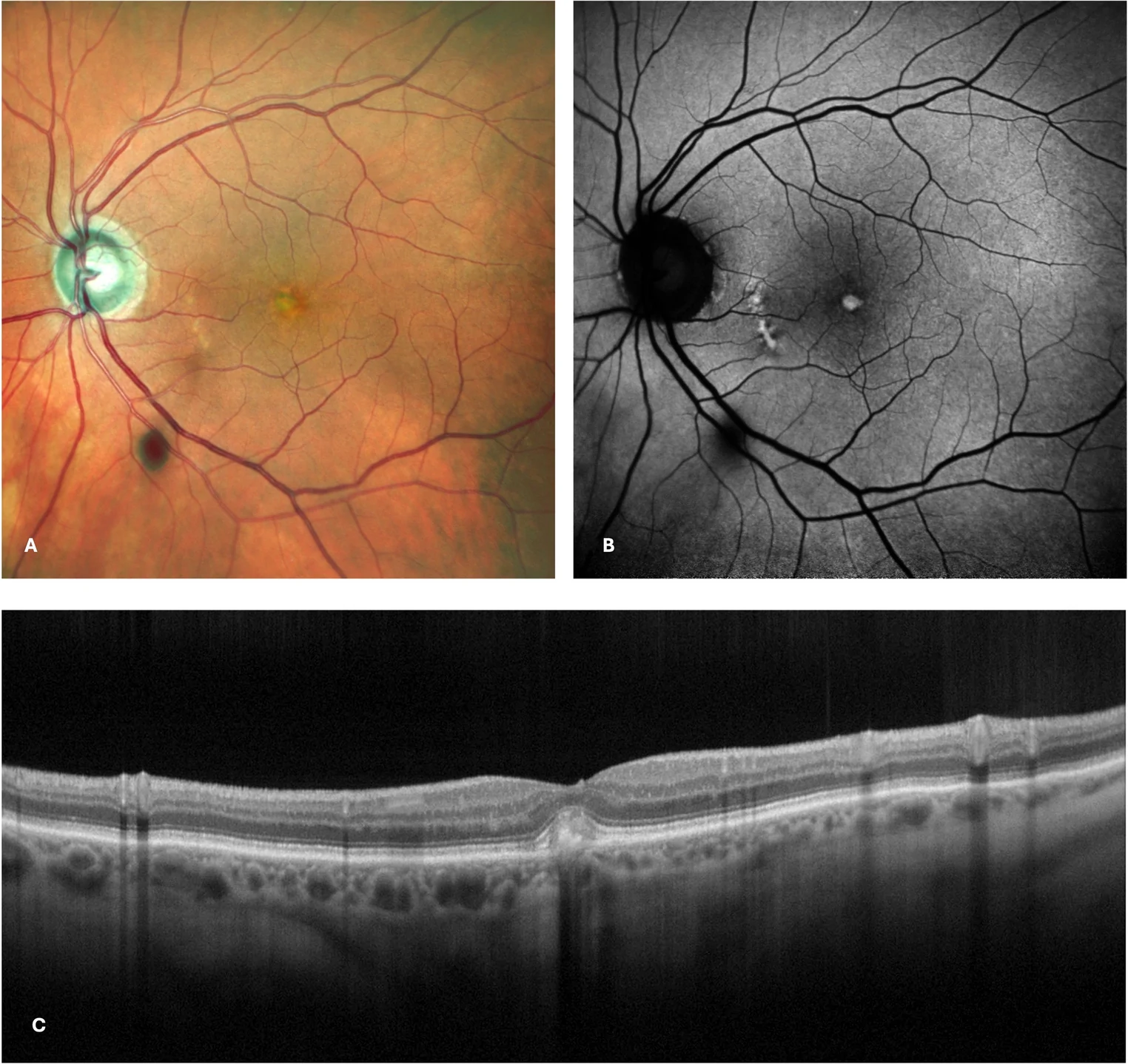
Multimodal imaging and acquired vitelliform lesion (AVL): Color fundus photography (CFP) shows a yellowish lesion in the foveal region (6 **A**), which appears hyperautofluorescent (6**B**) on fundus autofluorescence (FAF). Nasally few retinal pigment epithelium changes could be detected on CFP and FAF (6 **A** – **B**). Optical coherence tomography (OCT) B scan highlights the AVL as an hyperreflective lesion in the foveal region with focal interruption of the ellipsoid zone (EZ) (6** C**)

Upon examination with OCT, it corresponds to collected hyperreflective material between the photoreceptor layer and an irregularly shaped RPE band (Fig. 6). In some cases, this layer contains reflective puncta that resemble cellular structures [79]. 

Studies utilizing OCTA have observed that the buildup of vitelliform material leads to displacement of the capillary network, resulting in a reduction of the blood vessels’ density across the superficial capillary plexus and DCP, as well as within the choriocapillaris [80]. 

A large clinical cohort study demonstrated that AVLs are frequently associated with drusen, SDD, iHRF and PEDs [76, 81, 82]. Notably, the presence of SDDs and AVLs height have been identified as key risk factors for the progression of AVLs to complete RPE and outer retinal atrophy (RORA) [82]. 

The clinical evolution of AVLs has been further clarified in recent longitudinal studies. In a retrospective analysis of 163 eyes with AVL, 50% collapsed to atrophy, 38% remained stable, 10% resorbed, and 2% developed MNV over a mean follow-up period of 46 months [81]. 

The progression of AVLs can be further complicated by the development of MNV or foveal atrophy. A recent study aimed to investigate the evolution of AVLs in AMD eyes indicated that 12% of AVL cases resulted in type 1 MNV, while 44% evolved to atrophy [74, 81]. 

In acquired vitelliform macular dystrophy (AVMD), there are few similarities with AMD, involving the same population and being linked with drusen. However, a key distinction lies in the localization of lipofuscin accumulation: in AVMD, it occurs within the subretinal space, whereas in AMD, it accumulates beneath the RPE. Despite this difference, distinguishing between these two conditions can still pose a challenge due to overlapping features [78]. 

Moreover, SRF has been observed in cases of AVMD, a feature that could raise further concerns for a possible transition to exudative subtype. Interestingly, it is worth noting thatNNAMD may also exhibit SRF despite the absence of MNV [78]. 

Once excluded a MNV, the buildup of SRF alongside vitelliform lesions has been attributed to a mechanical displacement occurring between the RPE and the outer retinal layers. This displacement impedes the RPE ability to effectively pump out liquefied lipofuscin debris [76]. In these cases, the existing literature lacks a consensus regarding the efficacy of anti-VEGF (vascular endothelium growth factor) injections. Previous authors have proposed two approaches: closely monitoring the condition and opting for treatment only if there is an increase in SRF accompanied by symptomatic changes or administering an anti-VEGF injection and assessing the response within a two-week period [81, 83]. If the fluid dissipates following the injection, there is a high probability of MNV. Conversely, if the SRF remains stable or shows slight reduction, it can be inferred that the patient likely has AVMD.

## Outer retinal tubulations

Outer retinal tubulations (ORTs) represent a degenerative phenomenon involving the reorganization of the outer retina, primarily observed in eyes with macular disruption and absence of RPE. ORTs are common in advanced AMD, but they could be observed more rarely in other retinal disorders [84]. 

The term “tubulations” derived from their tubular morphology, which becomes apparent in frontal sections obtained by enface OCT scans, also known as C-scans [85]. 

ORTs appear on OCT as round hyporeflective formations, possibly containing a few focal hyperreflective spots, consistently surrounded by a hyperreflective band. The hyperreflective borders seem to represent a reorganization of photoreceptor and Müller cells, suggesting a reparative response or reaction to injury [84]. In NVAMD eyes, with clear evidence of vascular network, the hyporeflective ORT lumen can be erroneously identified as intraretinal or SRF, leading to potential misdiagnosis. Recognizing ORT as a distinct process independent of exudation is crucial to prevent unnecessary treatment interventions [84, 86, 87]. The characteristic aspect of ORTs supports the differential diagnosis with complete hyporeflective retinal cystoid lesions.

In the context of AMD, these tubulations are typically sited in close proximity to areas of neovascular fibrosis or retinal atrophy and at the level of the ONL [86]. 

Histological examination unveiled interconnected tubes comprising viable cone photoreceptors, intertwined with and enveloped by Müller glial processes [86].

As the RPE begins to atrophy, the ELM descent changes from flat to curved, then reflected to scrolled, and, finally, an area of ORT may appear [88]. 

The clinical significance of ORTs resides in their prognostic value, as they manifest in cases of advanced disruption of the outer retina, indicating poor visual function.

## Geographic atrophy (GA)

In the context of AMD, the term “macular atrophy” denotes the loss or irreversible thinning of macular tissue observed in eyes affected by both NNAMD and NVAMD.

**Fig. 7:**
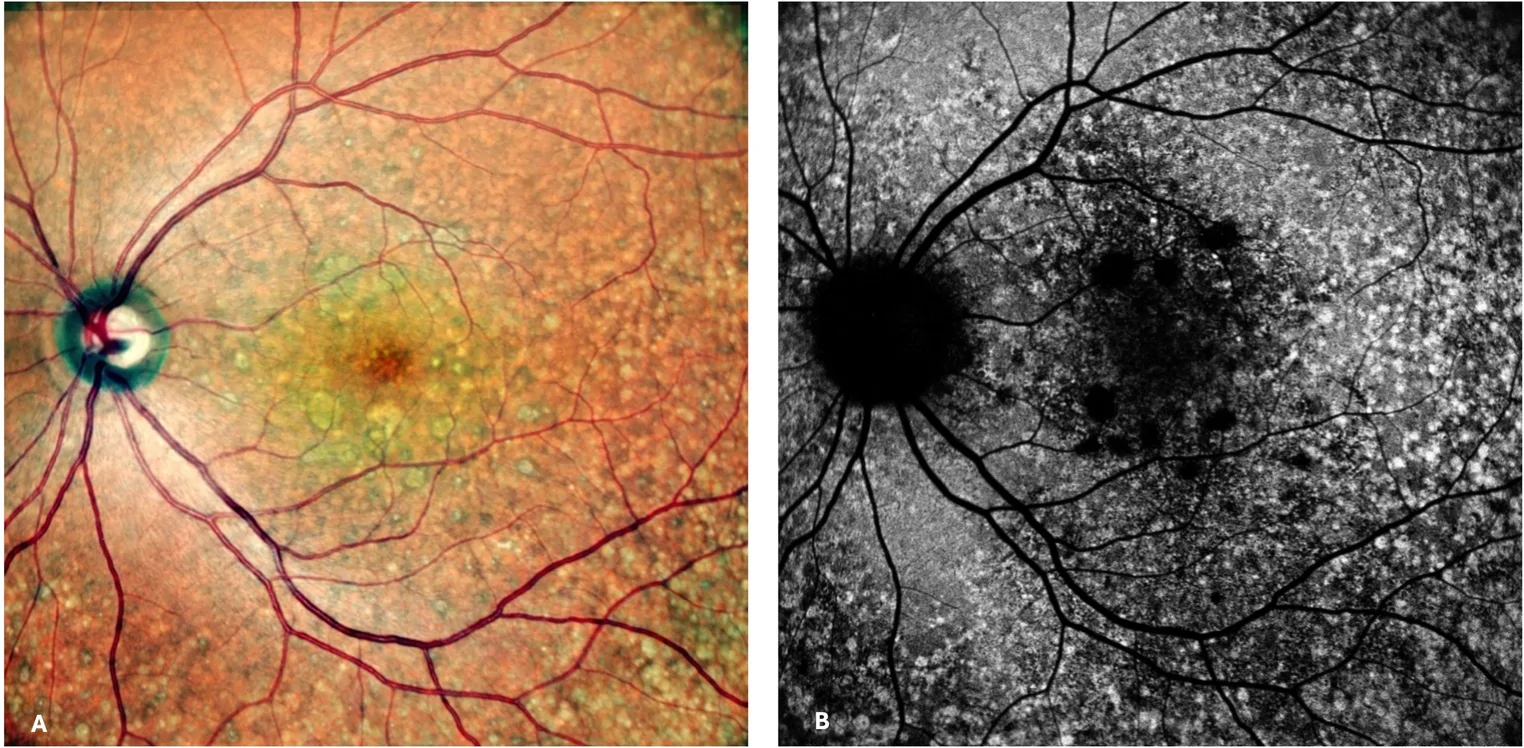
**Early geographic atrophy**: Color fundus photography (CFP) shows patchy areas of atrophy at the posterior pole with no involvement of the fovea, associated with widespread drusen (7 A). Fundus autofluorescence (FAF) reveals patchy hypoautofluorescent areas due to chorioretinal atrophy and hypo/hyperautofluorescent changes in correspondence to the diffuse drusen (7B)

**Fig. 8:**
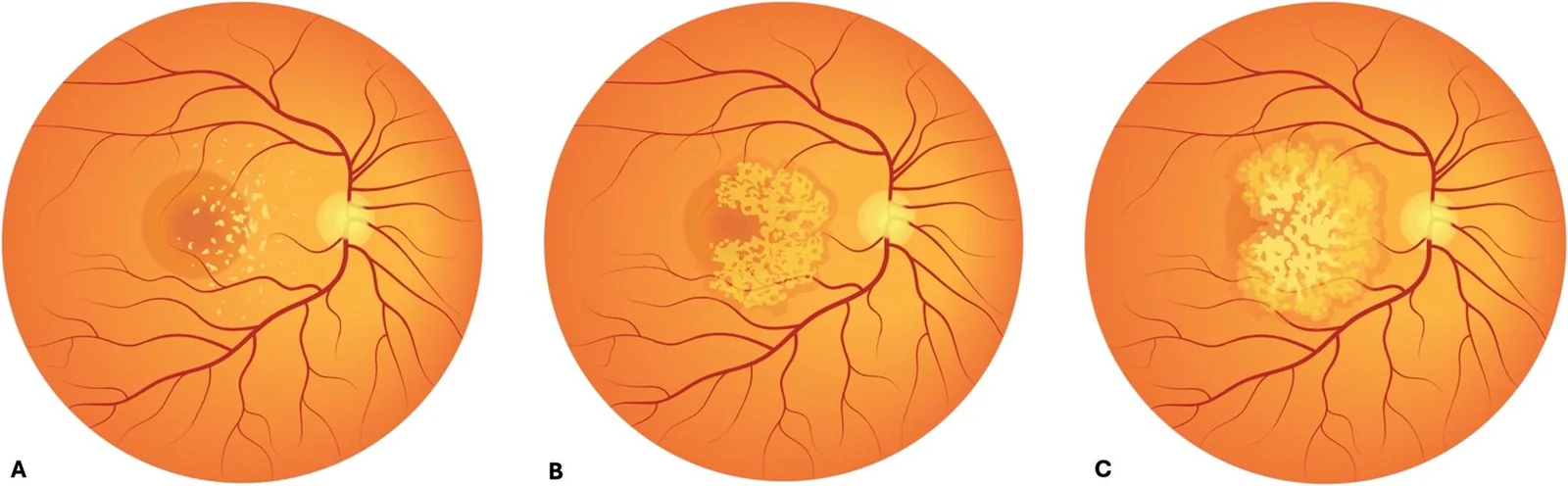
Iconographic representation of non neovascular age – related macular degeneration (NNAMD): The iconographic representation of NNAMD shows the progression of the disease from the early stage (8 **A**) with only few drusen at the posterior pole, towards an intermediate stage (8**B**), characterized by the development of outer retina and retinal pigment epithelium atrophy with foveal sparing, to the most advanced stage of geographic atrophy with foveal involvement (8 **C**). [Biorender Algorithm - Free Access - https://www.biorender.com/]

Traditionally, GA is defined on CFP as a sharply delineated circular or oval area exhibiting hypopigmentation or depigmentation, allowing visualization of choroidal vessels (Fig. 7). The atrophy tends to expand with a characteristic C – shape and to involve the fovea in the most advanced stage (Fig. 8).

The size requirements to define as GA vary among studies, ranging from 1/8 to 1/4 of a disc area (approximately 175 μm and 430 μm in diameter, respectively) on CFPs [89]. 

Nowadays, FAF still represents the gold standard method to diagnose GA and monitor its progression. FAF imaging reveals the inherent glow from lipofuscin and melanolipofuscin, which are metabolic leftovers building up in the RPE cells. The absence of RPE, beyond the outer retina layer and choriocapillaris, is the main responsible of FAF abnormalities. Indeed, the RPE cells degeneration is directly associated with lipofuscin disappearance and consequently with markedly reduced FAF signal over atrophic areas. It is therefore possible to clearly define the margins of atrophy, also considering the hyperautofluorescent margins that correspond to suffering, but still functional, adjacent cells. Since the fovea cannot be distinguished by the surrounding atrophic areas on FAF, owing to the presence of luteal pigment, exploring alternative imaging techniques such as near-infrared reflectance (NIR) or OCT is advisable for detecting foveal involvement.

NIR imaging can easily assess the foveal status, since it is not influenced by the luteal pigment, and furthermore, it could evaluate the extension of atrophy [90]. It also resulted to be comparable to FAF in GA lesions quantification, although in few cases the atrophic areas appear isoreflective in NIR, likely due to the choroidal thickness [91, 92]. In these cases, the OCT B scan, usually obtained simultaneously with NIR imaging, can confirm the presence of GA. Additional advantages of NIR are the greater availability of this modality and the less discomfort for the patients, compared to FAF.

Recently a consensus of medical retina experts (Classification of Atrophy Meeting, CAM, ) has provided two new fundamental concepts of retinal atrophy in NNAMD: complete RPE and outer retinal atrophy (cRORA) and incomplete RPE and outer retinal atrophy (iRORA) [93]. 

These novel concepts would be described in detail below in the present review.

**Fig. 9:**
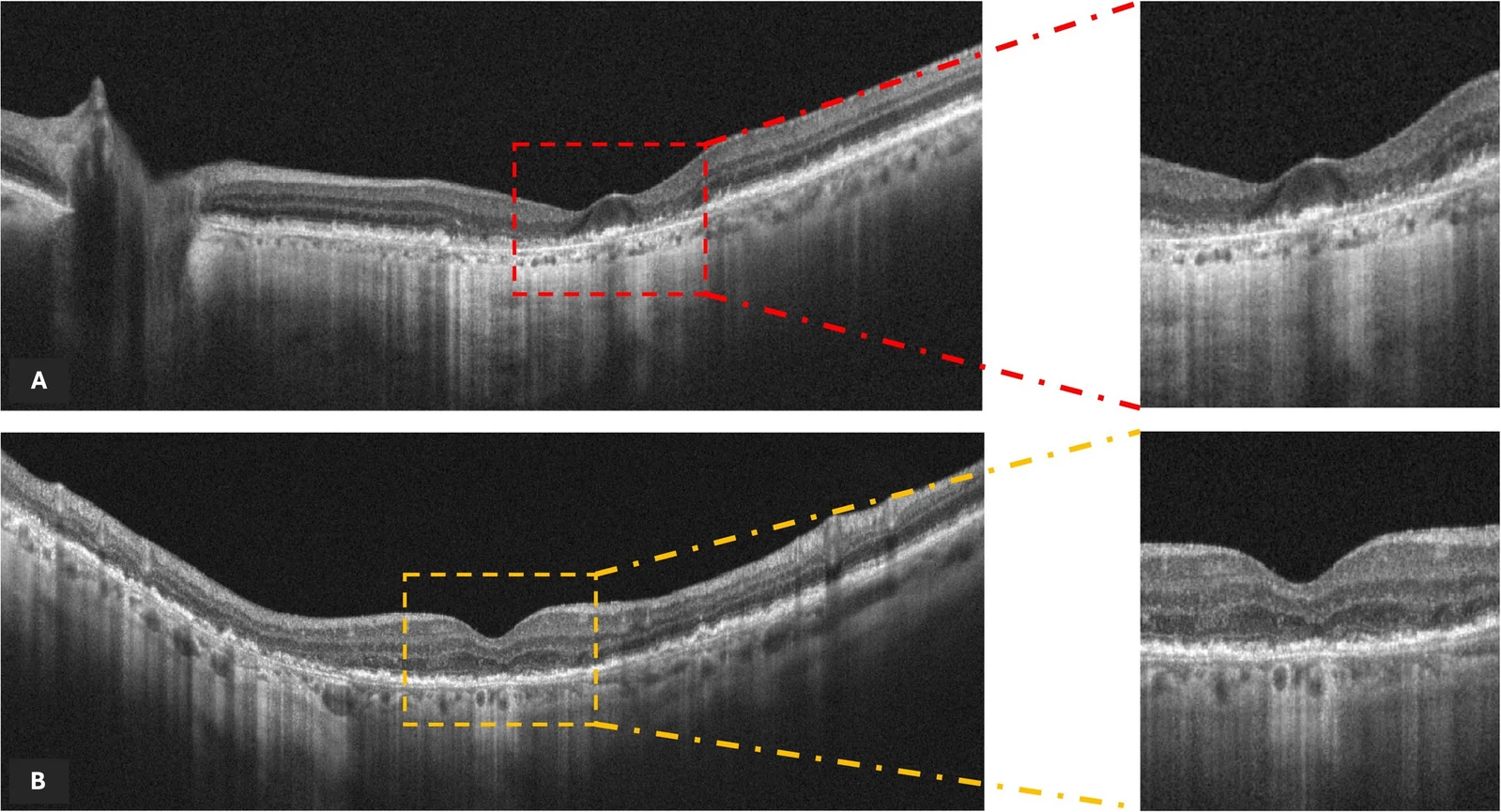
Optical coherence tomography (OCT) features in non neovascular age-related macular degeneration (NNAMD): OCT B – scan showing hyporeflective wedges in the macular region (9 **A**) and loss of integrity of external limiting membrane (ELM) and ellipsoid zone (EZ) (9**B**)

A thorough analysis of OCT scans reveals that several features may precede or be found contemporary to cRORA, such as inner nuclear layer (INL) and outer plexiform layer (OPL) subsidence, ONL thinning, hyporeflective wedge, loss of integrity of ELM and EZ, ELM descent and OCT signal hypertrasmission (Fig. 9).

It has also been demonstrated that choriocapillaris impairment on OCTA could be detected in the surrounding areas and moreover, it seems to manifest earlier than RPE changes on FAF [94, 95]. Additionally, in light of the possible co-existence of atrophy and MNV, OCTA should always be performed in order to guide the best therapeutical management.

The diameter of retinal vessels has been linked to the severity of AMD; specifically, smaller arterioles have been observed in eyes with GA, though they do not necessarily predict the onset of central GA [96]. 

Measurements of GA size using enface OCT have shown a good match with the decreased autofluorescence regions identified on FAF images, although OCT tends to report slightly smaller lesion sizes [97]. Velaga et al. found that the average GA size measured on FAF was 8.1 ± 5.04 mm^2^, whereas the initial automated measurement of GA by OCT showed a smaller average of 6.82 ± 3.84 mm^2^. However, after manually correction for segmentation errors in OCT, the GA size adjusted to 7.29 ± 4.18 mm^2^, significantly enhancing the correlation with the dimension determined by FAF [97]. 

Retromode is a comparatively new imaging technique that utilizes transillumination principles, capturing images through a scanning laser ophthalmoscope (SLO). Retromode utilizes a scanning laser ophthalmoscope with a laterally deviated aperture, either to the left (DL) or right (DR), instead of a central one, to capture images. This method allows the infrared laser light, which can penetrate deeper than visible light, to reach the outer layers of the retina, the RPE, and the choroid, enhancing the visualization of these structures.

**Fig. 10:**
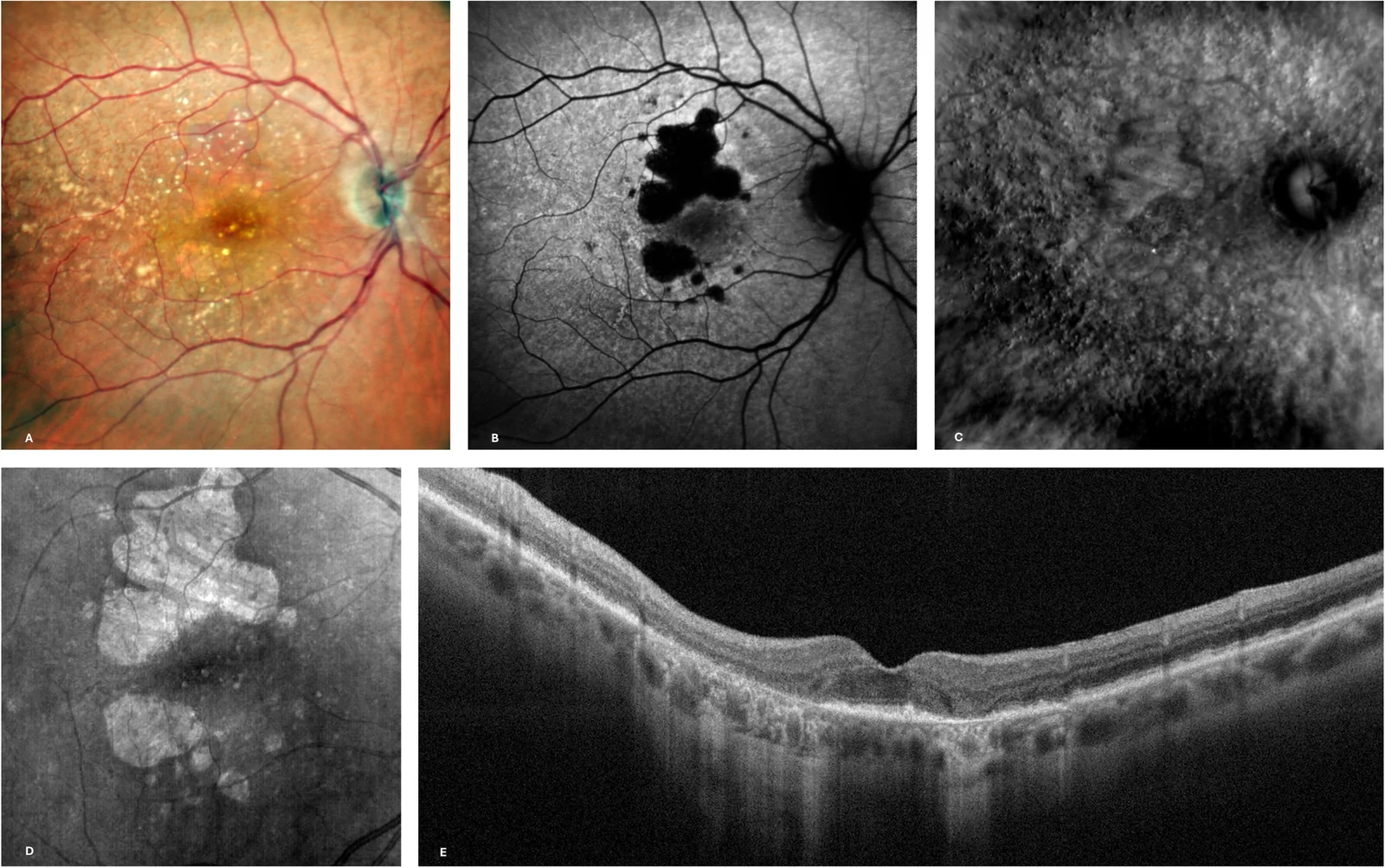
Multimodal imaging of geographic atrophy (GA) patient with foveal sparing. Color fundus photography (CFP) shows several soft drusen at the posterior pole associated with patchy areas of atrophy which allows to appreciate the underlying choroidal vessels (10 **A**). At fundus autofluorescence (FAF), hypoautofluorescent signal is evident in the areas of chorioretinal atrophy, with relevant foveal sparing (10**B**). The atrophic regions appear as slightly raised with well demarcated margins on retromode imaging (RM) and hyperreflective on enface optical coherence tomography (OCT) and infrared image (10 **C** – **D**). The OCT B scan shows clearly the foveal sparing by the atrophic process. Signal hypertrasmission can be appreciable in the perifoveal areas, due to retinal pigment epithelium (RPE) and outer retina atrophy (10**E**). Additionally, hyporeflective wedges could be observed at the margin of the residual ellipsoid zone and RPE layers (10**E**)

**Fig. 11:**
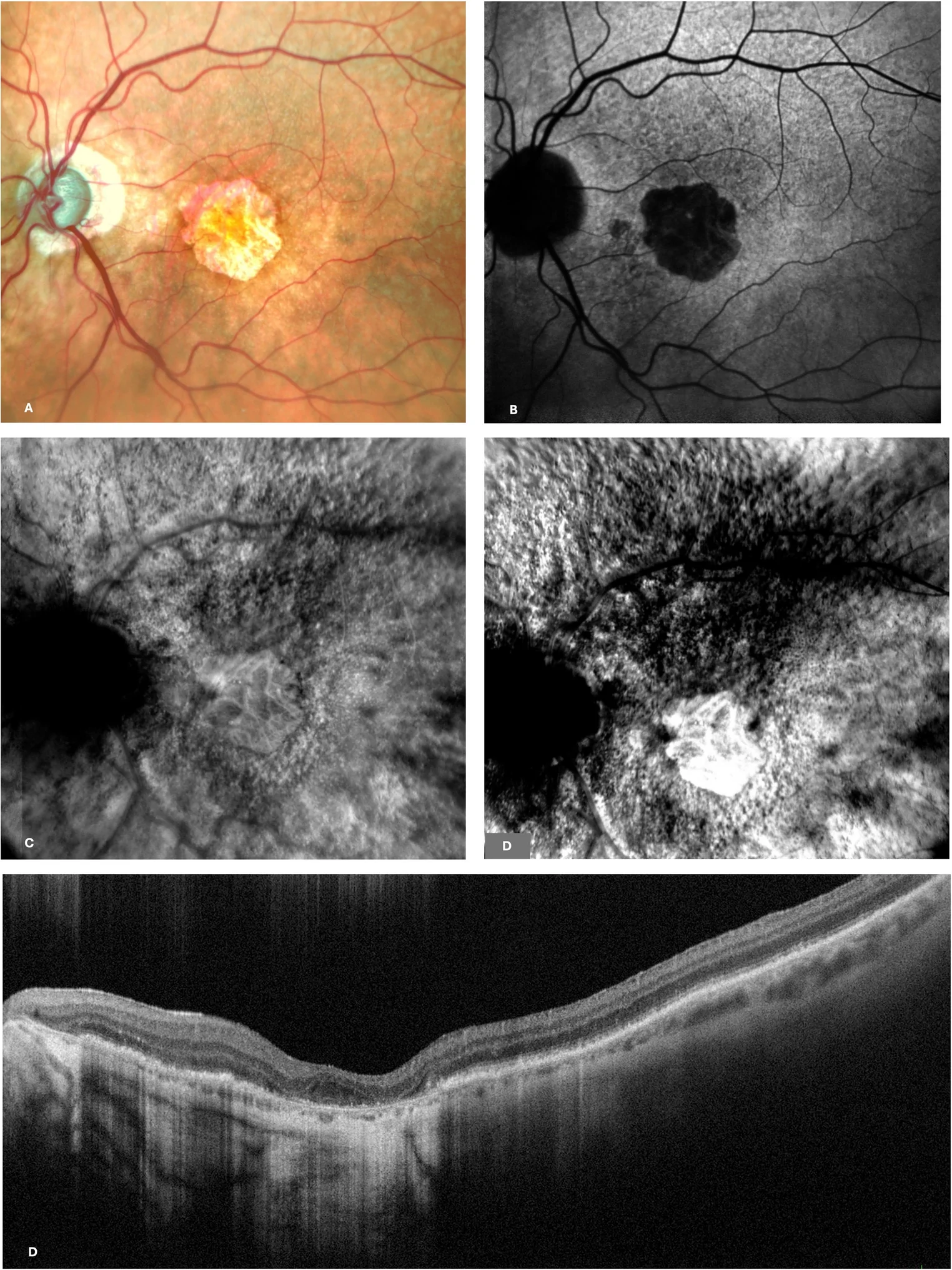
Multimodal imaging of geographic atrophy (GA) patient with foveal involvement: Color fundus photography (CFP) shows a sharply demarcated area of chorioretinal atrophy involving the fovea (11** A**), which appears hypoautofluorescent at fundus autofluorescence (FAF) (11**B**). The two retromode images (RM), obtained with right and left side opening provide different aspect of the atrophy, greyish and slightly depressed (11 **C**) and hyperreflective in the second image (11**D**). The optical coherence tomography (OCT) B scan shows clearly the foveal involvement by the atrophic process, with complete absence of retinal pigment epithelium (RPE) and outer retinal layers, with OCT signal hypertrasmission (11**E**)

Figures 10 and 11 show multimodal imaging of different stages of GA with foveal sparing and foveal involvement, respectively.

Corradetti et al.’s study compared this novel imaging technique to traditional methods for detecting and quantifying GA, showing that retromode resulted effective and consistent. According to their analysis, only minor statistically insignificant differences in GA area measurements between different methods were observed, suggesting that retromode may potentially have higher sensitivity to detect atrophic changes [98]. 

**Fig. 12:**
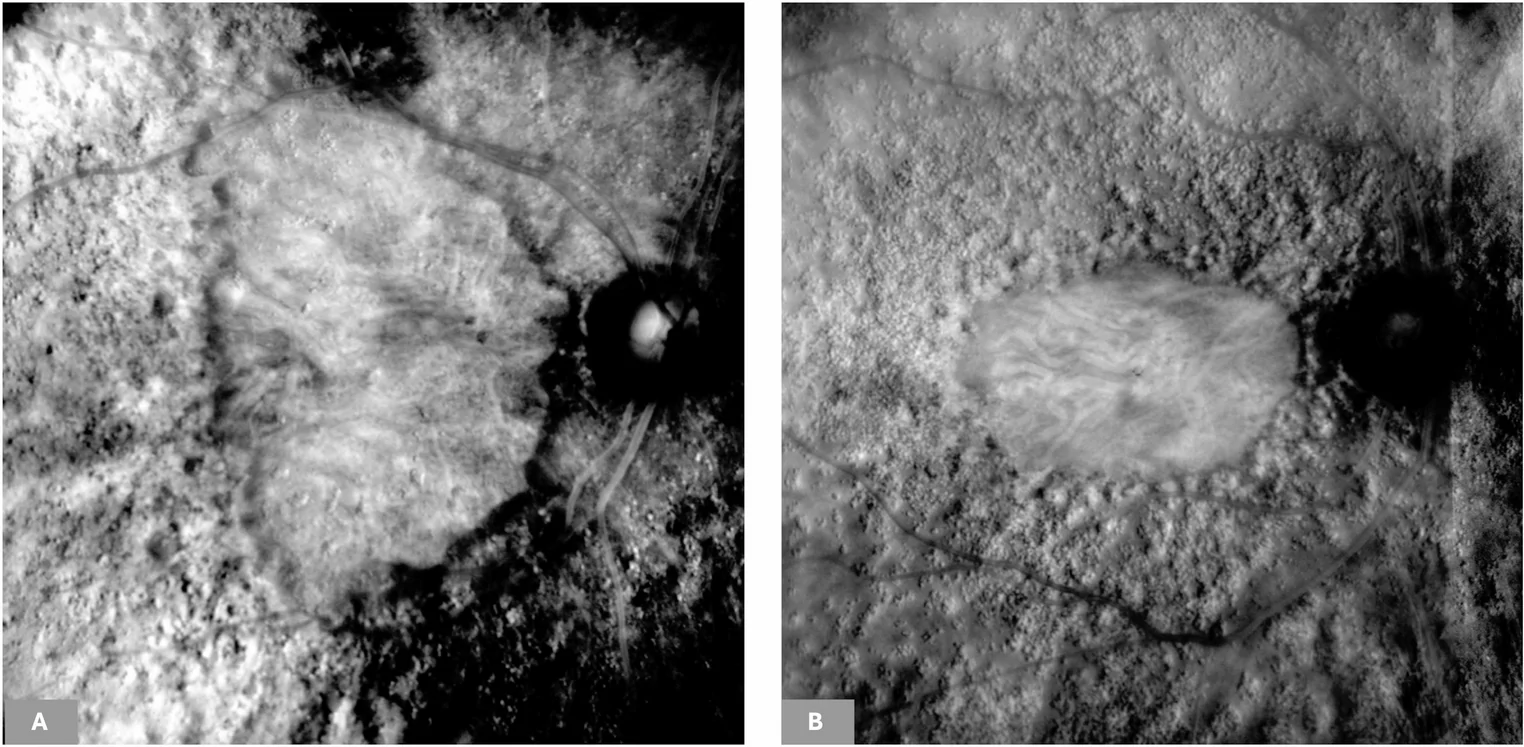
Retromode imaging of geographic atrophy (GA). Figures 12A and B show the typical hyperreflective aspect of GA on retromode imaging. Usually, the atrophic area appears slightly elevated due to the three-dimensional effect

Figure 12 illustrates clearly the classical appearance of GA on retromode.

However, retromode has not yet reached a significant diffusion worldwide and its use is mainly on a research level, it is therefore difficult to make definite conclusion.

Regarding GA progression, age stands out as the most common predictive factor, with multiple studies confirming an increase in GA severity with age [99–102]. 

Previous research has identified specific fundus features associated with high risk of progression to GA. The AREDS revealed that large drusen within the central macula and pigmentary changes observed on CFP confer an increased risk [103]. 

The occurrence of GA in one eye strongly suggests that the other eye will also be affected at some point. Although atrophy may not develop simultaneously, studies estimate a median time of 7 years between eyes progressing to GA, based on AREDS.

Lesion location also serves as a crucial indicator in GA progression, with extrafoveal lesions progressing more rapidly than foveal ones. This information aids clinicians in assessing the likelihood of disease progression for better patient education. The GA Study indicated that subjects with foveal-sparing GA at baseline experienced 2.8-fold faster lesion progression toward the periphery than toward the fovea. The median time for GA progression from initial discovery to foveal center involvement is estimated to be 2.5 years [104]. 

Lesion size is another standard metric used to evaluate GA severity and progression rate, with studies reporting lesion growth rates ranging from 0.53 to 2.6 mm^2^ per year. These rates vary based on both individual-specific factors and nonspecific (external) factors [104]. 

### Retinal pigment epithelium and outer retinal atrophy (RORA)

Recent advancements in multimodal imaging have opened an exceptional opportunity to delineate stages of AMD with greater precision than previously achievable. The CAM group proposed that atrophy in patients with NNAMD and NVAMD could be defined based on the affected anatomic layers, a characterization readily facilitated by OCT [105, 106]. 

Thus, to describe AMD related atrophy, the CAM group introduced four recommended terms: (1) cRORA, (2) iRORA, (3) complete outer retinal atrophy (cORA), and (4) incomplete outer retinal atrophy (iORA) [93]. These terms are applicable to describe atrophy with or without CNV.

**Fig. 13:**
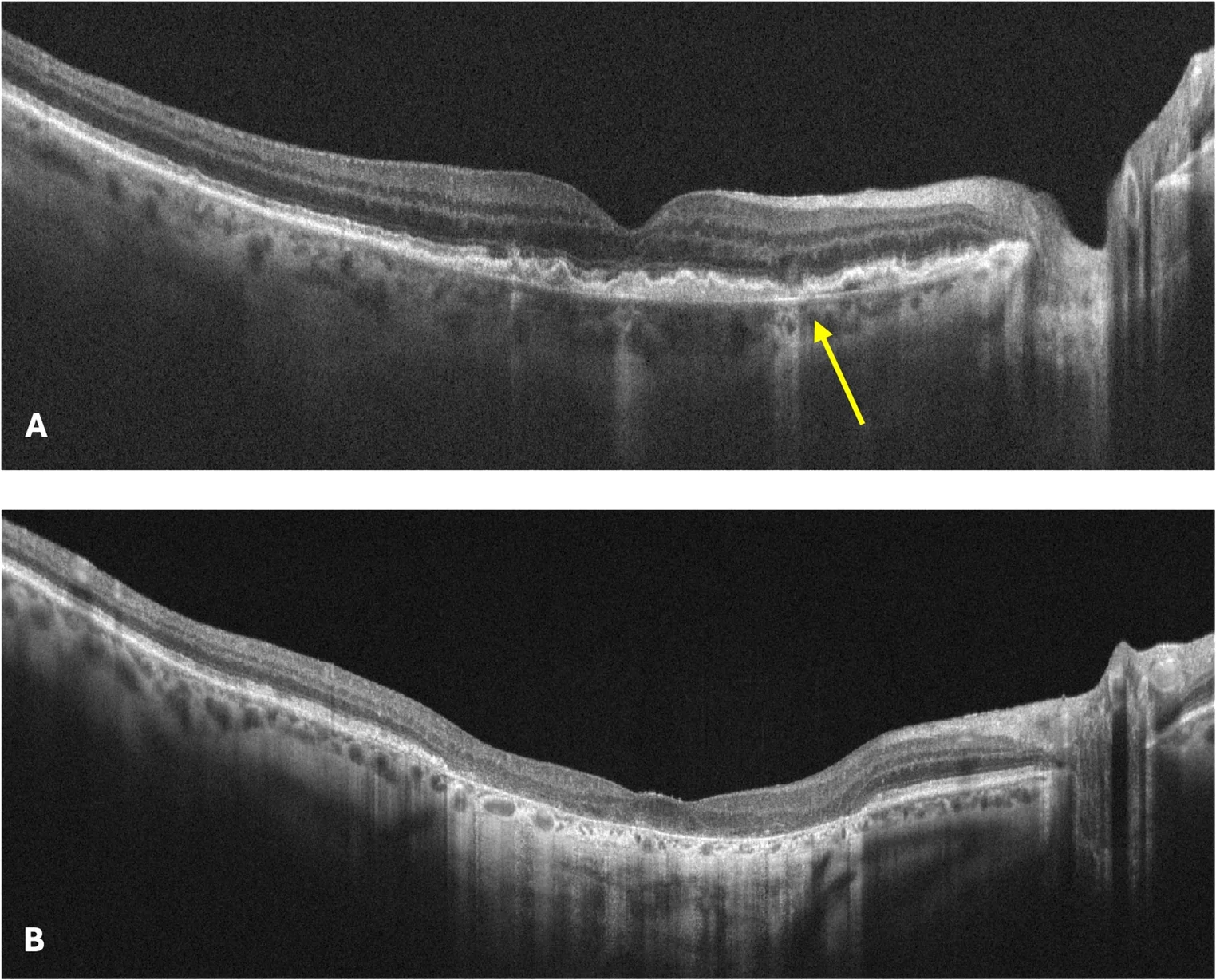
Incomplete retinal pigment epithelium (RPE) and outer retinal atrophy (iRORA) and complete RPE and outer retinal atrophy (cRORA). (**A**) The optical coherence tomography (OCT) B scan shows a typical case of iRORA, with signal hypertrasmission, disruption of the ellipsoid zone (EZ) and external limiting membrane (ELM), but still preserved RPE. Drusenoid pigment epithelium detachment (PED) can also be appreciated. (**B**) The OCT B scan represent a case of cRORA, with signal hypertrasmission > 250 micron, RPE disruption > 250 micron, photoreceptor degeneration and absence of RPE tear

iRORA, occurring alongside drusen, describes an area exhibiting choroidal signal hypertrasmission, a corresponding segment with attenuated or disrupted RPE, and degeneration of overlying photoreceptors. These characteristics have been identified as risk factors for progressing to cRORA, that is characterized by a region of hypertrasmission measuring at least 250 μm in diameter, an area of attenuated or disrupted RPE of at least 250 μm in diameter, evidence of associated photoreceptor degeneration, and absence of RPE tear. Figure 13 shows typical cases of iRORA and cRORA. The terms cORA and iORA are employed to denote disruption of the EZ and thinning of the outer retina, respectively, when the RPE remains intact [93, 105, 107]. 

Longitudinal investigations have revealed that ORA, devoid of RPE atrophy, is usually observable in eyes exhibiting SDD [108]. 

Nevertheless, participants in the CAM reached a consensus that the absence of RPE was consistently linked to thinning or loss of the outer retina in all instances [93]. 

Despite the term GA being firmly established in the literature for decades, the CAM group suggested retaining the term atrophy in absence of MNV (present or previous), when discernible on CFP. Hence, GA is considered a subset of the more comprehensive term cRORA, which encompasses macular atrophy with and without associated MNV. The term “nascent GA” is recommended for describing iRORA in the absence of MNV [93]. 

Assessment of iRORA and cRORA has demonstrated consistent inter-reader agreement, proving valuable not only in clinical settings but also in interventional trials for AMD. Wu et al. further asserted that iRORA serves as a significant risk factor for progression to GA, with the association likely driven by the development of specific features defining nascent GA, such as subsidence of the INL and OPL, hyporeflective wedges and OCT signal hypertrasmission [109, 110]. 

## Practical workflow for clinical assessment

The identification and documentation of imaging biomarkers in NNAMD should follow a systematic approach tailored to disease severity and risk profile. Based on the evidence reviewed herein, we propose the following practical framework for clinical assessment and monitoring.

For all patients with suspected or confirmed NNAMD, baseline assessment should include beyond visual acuity and fundus examination:

- CFP: assessment of drusen type, size, distribution and pigmentary changes.
- OCT: systematic evaluation for drusen characteristics, presence of SDD, iHRF (particularly in ONL), drusenoid PED, AVL, EZ/ELM integrity, and signs of iRORA or cRORA.
- FAF: assessment of RPE health, identification of hypo/hyperautofluorescent patterns, and detection/quantification of GA if present.

Additional imaging should be considered based on clinical findings:

- OCTA: if MNV is suspected (to differentiate drusenoid from vascularized PED) or to assess choriocapillaris integrity in eyes at high risk for disease progression.
- NIR: to better visualize SDD and to assess the foveal involvement if GA has already developed.

**Fig. 14:**
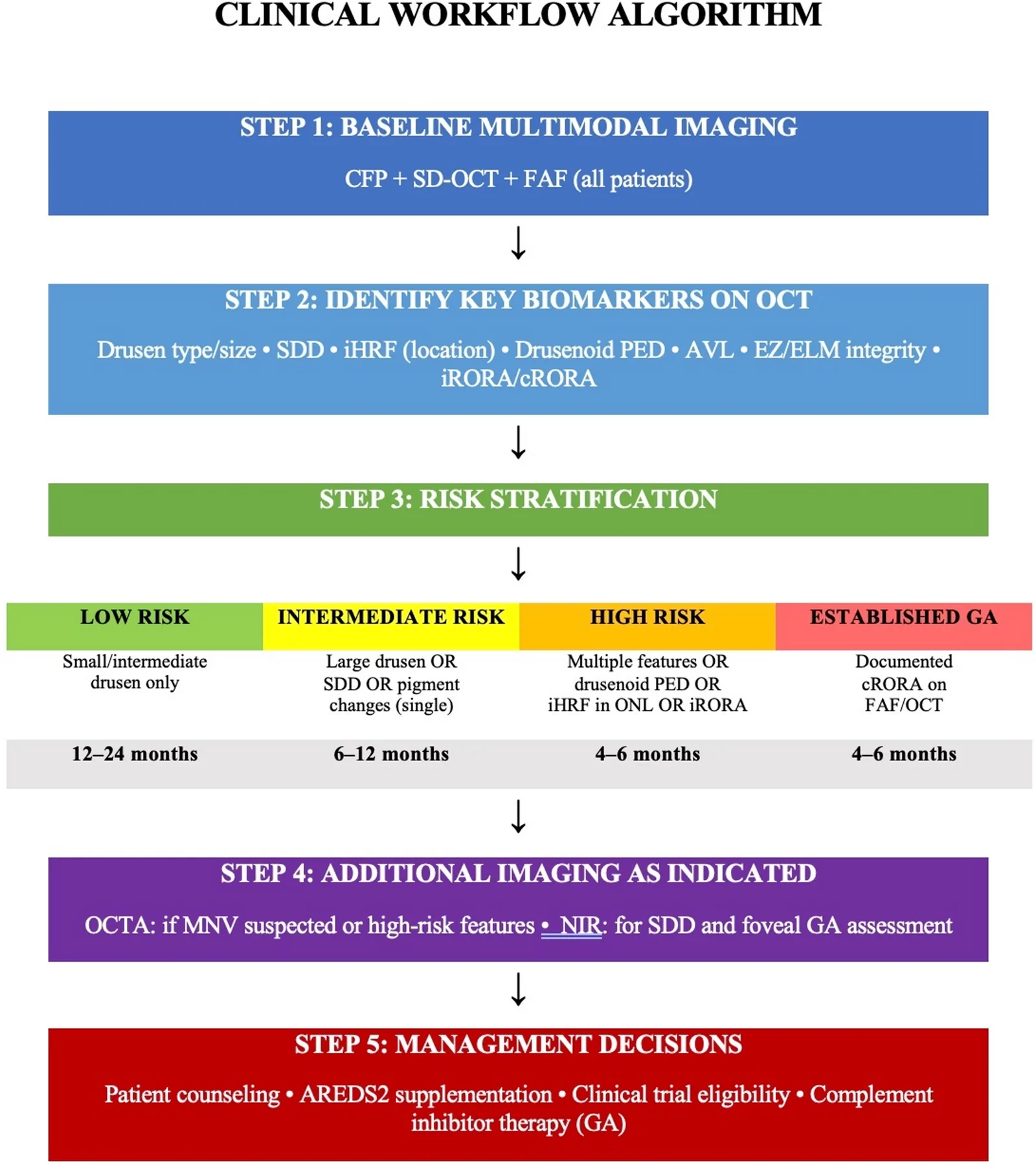
Clinical workflow algorithm for non-neovascular age-related macular degeneration (NNAMD) assessment and management. The figure represents a step-by-step guidance for NNAMD assessment at baseline and highlights the features that should be taken in consideration to estimate the risk of progression. Further, the follow up period is suggested, basing on the clinical/imaging findings

Follow-up frequency should be guided by the presence and combination of high-risk biomarkers (Table 1; Fig. 14). At each follow up, CFP, OCT and FAF should be performed in order to properly monitor the progression of the disease, with early detection of eventual changes and onset of atrophy.

**Table 1:** Suggested Follow-up Intervals Based on Risk Profile

Risk Category	Clinical/Imaging Features	Suggested Follow-up	Additional Recommendations
**Low Risk**	Small/intermediate drusen only; no pigmentary changes; no SDD	12–24 months	Patient education on Amsler grid monitoring; lifestyle counseling
**Intermediate Risk**	Large soft drusen OR SDD OR pigmentary abnormalities	6–12 months	Consider AREDS2 supplementation; reinforce self-monitoring
**High Risk**	Multiple high-risk features (large drusen + SDD + pigmentary changes) OR drusenoid PED OR iHRF in ONL OR iRORA	4–6 months	FAF at each visit; OCTA if MNV suspected; discuss clinical trial eligibility
**Established GA (cRORA)**	Documented GA on FAF/OCT	4–6 months	Quantify GA area (FAF or enface OCT); assess for foveal threat, discuss clinical trial eligibility

*Abbreviation: SDD* subretinal drusenoid deposits, *PED* pigment epithelial detachment, *iHRF* intraretinal hyperreflctive foci, *ONL* outer nuclear layer, *iRORA* incomple retinal pigment epithelium and outer retinal atrophy, *FAF* fundus autofluorescence, *OCTA* optical coherence tomography angiography, *MNV* macular neovascularization, *GA* geographic atrophy, *cRORA* complete retinal pigment epithelium and outer retinal atrophy, *OCT* optical coherence tomography

## Conclusion

Our knowledge of AMD has made significant steps forward in the last decades. Moreover, in the multimodal imaging era, novel biomarkers have been identified, allowing to have a holistic and specific evaluation of each patient. Furthermore, these new findings provide remarkable insights to monitor the disease and to predict its evolution.

Soft drusen, SDD, iHRF and drusenoid PEDs height resulted to be the most important and independent biomarker related to higher risk of progression of the disease to advanced stages.

Table 2 summarizes the main characteristic of the most relevant NNAMD biomarkers.

**Table 2:** Summary of Key Imaging Biomarkers in Non-Neovascular AMD

Biomarker	Definition / OCT Criteria	CFP Appearance	FAF Appearance	OCT Appearance	OCTA Findings	Prognostic Implication
**Hard Drusen**	< 63 μm diameter; focal Bruch’s membrane deposits	Small, yellow-white, discrete margins	Iso- to mildly hyperautofluorescent	Small dome-shaped RPE elevations; intact EZ/ELM	Minimal flow changes	Low risk if isolated; higher risk if numerous and clustered
**Soft Drusen**	> 125 μm diameter; basal linear deposits (BLinD)	Large, pale-yellow, indistinct borders	Variable hypo-/hyperautofluorescent	Dome-shaped RPE elevations with sloping sides (30–1000 μm)	Focal choriocapillaris flow deficits	High risk for progression to GA or MNV; larger volume correlates with higher risk of progression
**Cuticular Drusen**	25–75 μm; “saw-tooth”	Numerous small, round, clustered lesions	“Stars in the sky” on FA; central hypoAF with hyperAF rim	Saw-tooth RPE undulations	Variable	Phenotypes 2–3 associated with GA progression
**Subretinal Drusenoid Deposits (SDD)**	Subretinal deposits above RPE; Zweifel stages 1–4	Yellowish-white reticular pattern; best seen in blue light	Hypoautofluorescent spots with normal surrounding signal	Hyperreflective material between RPE and EZ; conical in advanced stages	Choriocapillaris flow impairment	Independent risk factor for late AMD; associated with type 3 MNV; poor treatment response
**Drusenoid PED**	≥ 350 μm; confluent soft drusen elevating RPE	Pale yellow/white elevated lesion; posterior pole	Variable; halo of decreased AF at margins	Smooth RPE elevation; moderate-high reflectivity material beneath	No vascular network (it is mandatory to exclude MNV)	42% progress to advanced AMD at 5 years; height predicts GA, width predicts MNV
**Intraretinal HRF (iHRF)**	Discrete hyperreflective foci; migrated RPE cells	Hyperpigmented spots	May correlate with hyperAF foci	Hyperreflective foci in ONL (highest risk) or inner layers	N/A	Strongest independent predictor of progression to GA; ONL location most significant
**Acquired Vitelliform Lesion (AVL)**	Subretinal hyperreflective material above RPE; ~1/3 disc diameter	Yellow, slightly elevated lesion, often subfoveal	Hyperautofluorescent	Hyperreflective material between photoreceptors and RPE	Reduced vessel density in SCP, DCP, choriocapillaris	50% collapse to atrophy; 2–12% develop MNV; height and SDD presence predict cRORA
**iRORA (Nascent GA)**	Incomplete RORA; hypertrasmission + RPE attenuation + photoreceptor loss; <250 μm	Not reliably visible	Subtle hypo-/hyperautofluorescent changes	Choroidal hypertrasmission; attenuated RPE; EZ/ELM disruption	Choriocapillaris dropout	Precursor to cRORA; identifies eyes at imminent risk of GA development
**cRORA (Geographic Atrophy)**	≥ 250 μm hypertrasmission; ≥250 μm RPE loss; photoreceptor degeneration; no RPE tear	Sharply demarcated depigmentation; visible choroidal vessels	Well-defined hypoautofluorescence; hyperAF margins	Complete RPE and outer retinal loss; hypertrasmission; INL/OPL subsidence	Choriocapillaris loss within and surrounding lesion	Irreversible vision loss; mean growth 0.53–2.6 mm²/year; median 2.5 years to foveal involvement

Abbreviations: *AF* autofluorescence, *BLinD* basal linear deposit, *CFP* color fundus photography, *DCP* deep capillary plexus, *ELM* external limiting membrane, *EZ* ellipsoid zone, *FA* fluorescein angiography, *FAF* fundus autofluorescence, *GA* geographic atrophy, *HRF* hyperreflective foci, *INL* inner nuclear layer, *MNV* macular neovascularization, *OCT* optical coherence tomography, *OCTA* OCT angiography, *ONL* outer nuclear layer, *OPL* outer plexiform layer, *PED* pigment epithelial detachment, *RORA* retinal pigment epithelium and outer retinal atrophy, *RPE* retinal pigment epithelium, *SCP* superficial capillary plexus, *SDD* subretinal drusenoid deposits

In light of novel available therapies and future drugs, a multimodal imaging approach will play a remarkable role to demonstrate their safety and efficacy.

## Method of literature search

To prepare this narrative review, a non-systematic PubMed search was performed between September 2023 and January 2025 to identify relevant literature on NNAMD and associated imaging biomarkers. Searches included combination of terms related to disease features, imaging biomarkers and therapeutic approaches. Articles published from 1990 to 2025 were considered.

Various study types were evaluated including review, systematic review, metanalysis, original research and large case series, with priority given to studies providing clinically meaningful or methodologically robust data. Articles were screened basing on title and abstract and included prior full-text reading for those deemed relevant. ClinicalTrials.gov was also consulted for trial updates and unpublished trial results.

## Data Availability

No datasets were generated or analysed during the current study.
